# Transcriptomic signatures in Gaucher disease subtypes: A systems biology perspective

**DOI:** 10.1016/j.ymgmr.2025.101274

**Published:** 2025-11-13

**Authors:** Mohammad Elahimanesh, Reza Ganjali, Mohammad Najafi

**Affiliations:** a Clinical Biochemistry Department, Faculty of Medical Sciences, Iran University of Medical Sciences, Tehran, Iran; b Microbial Biotechnology Research Center, Iran University of Medical Sciences, Tehran, Iran

**Keywords:** Gaucher disease, Pathway, Differential gene expression, Subtype, Network

## Abstract

Gaucher disease (GD) is a lysosomal storage disorder caused by the failure of *GBA1* (Glucosylceramidase Beta 1). The aim of study was to analyze and enrich signaling pathways with transcriptomic profiles in cultured skin fibroblasts of GD subtypes (GD1, GD2, GD3) using GEO datasets. Differentially expressed genes (DEGs) were identified using the Limma package in R with a significance threshold (adjusted *p*-value <0.05 and |log2FC| > 1) and were used in gene networks constructed by Cytoscape. In GD1, up regulated genes (*TP53*, *COL4A1*) were found in mRNA splicing and ECM organization, while down regulated genes (*IL6*, *TGFBR2*) were linked to cytokine and TGF-β signaling pathways. GD2 showed upregulation of *MMP1*, *CXCL8*, and interferon-related genes (*ISG15*, *MX1*), associated with interleukin-4/13 signaling and ECM dysregulation, reflecting severe neuroinflammation. GD3 exhibited upregulation of *FOS*, *AKT1* (>5 years), and *POSTN*, *EGR2* (<5 years), found in PI3K/AKT and myelination signaling pathways, alongside down regulated *CXCL8* and *PTGS2* linked to receptor tyrosine kinase signaling pathway. Gene networks identified hub genes (*PSAP*, *CTSB* for GD1; *LAMP2*, *GAA* for GD2/GD3) and enriched pathways (glycosphingolipid metabolism, lysosomal function). Top 5 % DEGs, including KDM5D (GD1), MMP1 (GD2), and FOSB (GD3), were proposed as subtype-specific markers. The findings highlighted distinct molecular signatures between GD1 (immune/ECM-focused) and GD2/GD3 (neuroinflammatory/neurodevelopmental), informing targeted diagnostics.

## 1 Introduction

Gaucher disease (GD) is an autosomal recessive lysosomal storage disorder caused by mutations in the *GBA1*, leading to deficient activity of the enzyme Glucosylceramidase Beta 1 [1]. It results in the accumulation of glucosylceramide within multiple organs, contributing to systemic and neurological manifestations [2,3]. GD is classified into three clinical subtypes: non-neuronopathic type 1 (GD1), acute neuronopathic type 2 (GD2), and chronic neuronopathic type 3 (GD3). While GD1 primarily affects visceral organs and the skeletal system, GD2 and GD3 exhibit varying neurological degrees, ranging from rapid neurodegeneration in GD2 to a more protracted course in GD3 [[4], [5], [6]].

Recent transcriptomic and proteomic studies have provided insights into the molecular landscape of GD, highlighting the involvement of immune dysregulation, lysosomal dysfunction, and neuroinflammation [7,8]. Some studies have reported changes in cytokine profiles in GD patients, upregulating pro-inflammatory mediators such as interleukin-6 (*IL-6*), tumor necrosis factor-alpha (*TNF-α*) and implicating chronic inflammation as a key contributor [9,10]. Additionally, dysregulation of extracellular matrix (ECM) components has been linked to bone abnormalities in GD1, whereas defects in neurotrophic signaling and apoptotic pathways have been associated with neuronal loss in GD2 and GD3 [11,12].

Given the clinical heterogeneity of GD, a deeper understanding of subtype-specific molecular perturbations is essential for refining therapeutic strategies. In this study, a comprehensive transcriptomic analysis of GD1, GD2, and GD3 was performed using publicly available gene expression datasets (GEO). Differentially expressed genes (DEGs) were identified and subjected to protein-protein interaction (PPI) network analysis, pathway enrichment, and cellular component localization to elucidate subtype-specific molecular signatures. Furthermore, we investigated whether age stratification within GD1 and GD3 reveals distinct gene expression patterns that may contribute to disease progression and severity. The results might enhance our understanding of the molecular basis of GD subtypes, emphasizing crucial pathways and uncover subtype- and age-specific transcriptomic signatures, offering novel insights into potential screening markers, and underscore the importance of personalized therapeutic approaches targeting specific disease mechanisms.

## 2 Materials and methods

### 2.1 Data acquisition and selection

To explore gene expression profiles associated with GD subtypes, publicly available transcriptomic datasets were retrieved from the Gene Expression Omnibus (GEO) database (https://www.ncbi.nlm.nih.gov/geo/). The search was conducted using a comprehensive set of keywords, including: (“*GBA*1” OR “ Glucosylceramidase Beta 1 “ OR “beta-glucosidase” OR “acid beta-glucosidase” OR “glucocerebrosidase” OR “glucocerebrosidase deficiency” OR “autosomal recessive deficiency of acid beta-glucosidase” OR “*GBA* mutation” OR “*GBA* deficiency” OR “Gaucher disease” OR “Gaucher's disease”) in combination with study type filters (“Expression Profiling by Array” OR “Expression Profiling by High-Throughput Sequencing”) and data limitation to *Homo sapiens* up to 2024. Based on these criteria, two relevant datasets were identified and selected: GSE21899, which included samples from GD1 and GD3, and GSE124283, which focused on GD2 (Table 1).

**Table 1:** Datasets used in the study.

GEO	Sample	Gender	Status	Mutation	Age	Platform
GSE21899	GSM544727	Male	Wild-Type Control		29	GPL571
	GSM544728	Female	Wild-Type Control		25	
	GSM544729	Female	Wild-Type Control		32	
	GSM544730	Female	Wild-Type Control		22	
	GSM544731	Male	Gaucher Disease Type1	N370S	68	
	GSM544732	Male	Gaucher Disease Type1	N370S	71	
	GSM544733	Male	Gaucher Disease Type1	N370S	47	
	GSM544734	Female	Gaucher Disease Type1	N370S	42	
	GSM544735	Male	Gaucher Disease Type1	N370S	31	
	GSM544736	Male	Gaucher Disease Type3	L444P	4	
	GSM544737	Female	Gaucher Disease Type3	L444P	2	
	GSM544738	Male	Gaucher Disease Type3	L444P	4	
	GSM544739	Female	Gaucher Disease Type3	L444P	25	
	GSM544740	Male	Gaucher Disease Type3	L444P	4	
GSE124283	GSM3526881	Male	Wild-Type Control		NA	GPL10904
	GSM3526884	Male	Wild-Type Control			
	GSM3526899	Female	Wild-Type Control			
	GSM3526902	Female	Wild-Type Control			
	GSM3526911	Male	Wild-Type Control			
	GSM3526914	Female	Wild-Type Control			
	GSM3526923	Male	Wild-Type Control			
	GSM3526926	Female	Wild-Type Control			
	GSM3526929	Male	Wild-Type Control			
	GSM3526932	Female	Wild-Type Control			
	GSM3526947	Male	Wild-Type Control			
	GSM3526950	Male	Wild-Type Control			
	GSM3526959	Female	Wild-Type Control			
	GSM3526962	Female	Wild-Type Control			
	GSM3526975	Male	Wild-Type Control			
	GSM3526992	Male	Wild-Type Control			
	GSM3526995	Male	Wild-Type Control			
	GSM3526998	Female	Wild-Type Control			
	GSM3527010	Female	Wild-Type Control			
	GSM3527020	Male	Wild-Type Control			
	GSM3526988	Female	Gaucher Disease Type2	NA		
	GSM3527004	Male	Gaucher Disease Type2	NA		
	GSM3527007	Female	Gaucher Disease Type2	NA		
	GSM3527008	Female	Gaucher Disease Type2	NA		

**NA**, Not Available.

### 2.2 Datasets

GSE21899 dataset contained 14 samples (4 control, 5 GD1, and 5 GD3) (GPL571). For GD1, samples were stratified by age into two groups: <50 years [*n* = 3] and > 50 years [*n* = 2]) vs. 4 controls. For GD3, samples were stratified by age into two groups: <5 years [*n* = 4] and > 5 years [*n* = 1]) vs. 4 controls. GSE124283 dataset contained 24 samples (20 control, 4 GD2) (GPL10904). All expression data were derived from cultured GD skin fibroblasts as provided in the GEO records and are summarized in Table 1.

### 2.3 Differential expression genes (DEGs)

For each subtype, Batch effects were minimized using the sva R package, followed by quantile normalization and log2 transformation. DEGs were identified by comparing patient samples with their respective control groups based on Log Fold Changes (LogFCs). The statistical significance of differential expression was assessed using the Limma package, applying empirical Bayes moderation. Transcriptomic analyses were performed with control for false discovery rate (FDR), applying the Benjamini-Hochberg method for statistical correction. The criteria for DEGs included; up regulated genes: adjusted *p*-value <0.05 and log2 fold change (LFC) > 1, and down regulated genes: adjusted p-value <0.05 and LFC < −1. Heatmaps were generated using the pheatmap package in R. Genes were clustered based on gene expression Log values across sample groups (Euclidean distance, complete linkage).

### 2.4 Gene network

For DEG sets (up regulated and down regulated genes in each subgroup), gene networks were constructed using the STRING v12.0 database (https://string-db.org/) with the following parameters: Active interaction sources: Databases, Experiments, Text-mining; Minimum required interaction score: 0.7; Disconnected nodes hidden. Networks were visualized in Cytoscape, with node centrality metrics (degree, betweenness) calculated via the Network Analyzer tool. In each network, nodes exhibiting both high connectivity (a greater number of edges with other nodes) and significant fold change magnitudes were designated as hub genes within biological networks.

### 2.5 Network enrichment

To identify biological processes, Gene Ontology (GO) and Reactome pathway enrichment analyses were performed using Cytoscape with the ClueGO v2.5.10 plugin [13] (Enrichment significance: *p* < 0.05; Kappa score threshold: 0.4 for term grouping) on gene networks stratified with age subgroups.

### 2.6 Potential gene screening markers

A percentile stratification based on their log2FC values were estimated for screening of GD. Genes within the top 5 % (>95th percentile) were presented as subgroup-specific marker candidates forming subtype- and age-specific panels.

### 2.7 Statistical and computational considerations

All analyses were performed in R (version 4.4.2) and Cytoscape (version 3.9.1). Differential gene expression analysis was performed using Limma [14] and sva packages [15], with batch effect correction applied via ComBat. All transcriptomic analyses, including differential expression genes (DEGs), protein-protein interaction (PPI) networks, and enrichment analyses, were conducted using the false discovery rate (FDR) with the Benjamini-Hochberg correction.

## 3 Results

### 3.1 Gaucher disease type 1 (GD1)

#### 3.1.1 Patients older than 50 years age

##### 3.1.1.1 Down regulated genes

**Fig. 1:**
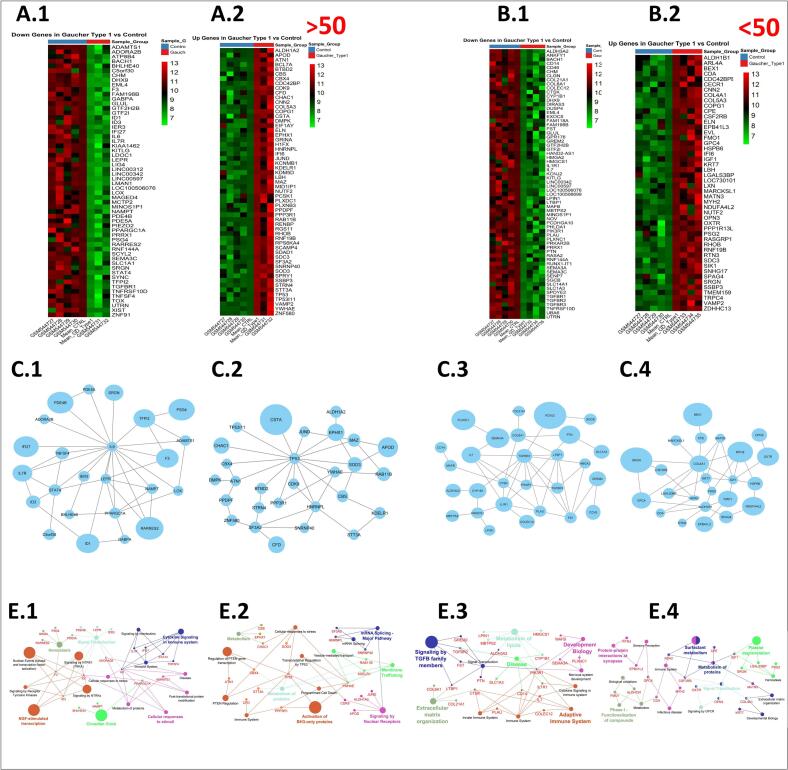
Enriched up and down regulated gene networks in Gaucher type 1. The down and up regulated genes among patients older than 50 years (A.1: Down regulated genes and A.2: Up regulated genes) and younger than 50 years (B.1: Down regulated genes and B.2: Up regulated genes). PPI networks and Reactome pathway enrichments in patients older than 50 years (C.1 and E.1, Down regulated DEGs; C.2 and E.2, Up regulated DEGs). PPI networks and Reactome pathway enrichments in patients younger than 50 years (C.3 and E.3, Down regulated DEGs; C.4 and E.4, Up regulated DEGs).

A heatmap was generated to visualize the lowly expressed gene profile across patient and control samples (Fig. 1, A.1). The downregulated DEGs (*n* = 58), identified by LogFC analysis between patient and control samples, were used to construct a PPI network containing 24 nodes and 42 edges (protein-protein interaction enrichment, *p* = 1.21e-4). The key hub genes included *IL6, LEPR, PPARGC1 A, STAT4, IL7R, TFPI2, ADAMTS1*, and *NAMPT* (Fig. 1, C.1). Functional enrichment highlighted cytokine-mediated immune signaling (*p* = 1.01e-2), cellular response to stimuli (*p* = 0.3e-3), immune system (*p* = 1.31e-4), signaling by interleukins (*p* = 0.21e-5), *NGF*-stimulated transcription (*p* = 1.00e-2), and *TRKA* signal transduction (*p* = 0.9e-3) (Fig. 1, E.1). Cellular component analysis localized these genes to cytoplasm and intracellular organelles (Supplementary, Fig. 1A).

##### 3.1.1.2 Up regulated genes

A heatmap visualized log-transformed expression profile of highly expressed genes across patient and control samples (Fig. 1, A.2). Differential expression analysis identified 57 up regulated genes based on the LogFC analysis between patient and control samples, applied to construct network (28 nodes and 40 edges). The key hub genes containing *TP53, HNRNPL, SF3A2, YWHAE, ATN1, SOD3, MAZ,* and *CBS* were identified within the network. The protein-protein interaction (PPI) network contained 28 nodes and 40 edges (protein-protein interaction enrichment, *p* = 8.66e-4) (Fig. 1, C.2). The network enrichment revealed significant involvement in mRNA splicing (*p* = 0.8e-3), membrane trafficking (*p* = 1.7e-4), protein metabolism (*p* = 0.2e-1), nuclear receptor signaling (*p* = 0.05e-3), *PTEN* regulation (*p* = 0.6e-5), and activation of BH3-only proteins (*p* = 3.1e-4) (Fig. 1, E.2). These genes were predominantly localized to vesicles, extracellular space, and plasma membrane (Supplementary, Fig. 1B).

#### 3.1.2 Patients younger than 50 years age

##### 3.1.2.1 Down regulated genes

**Fig. 2:**
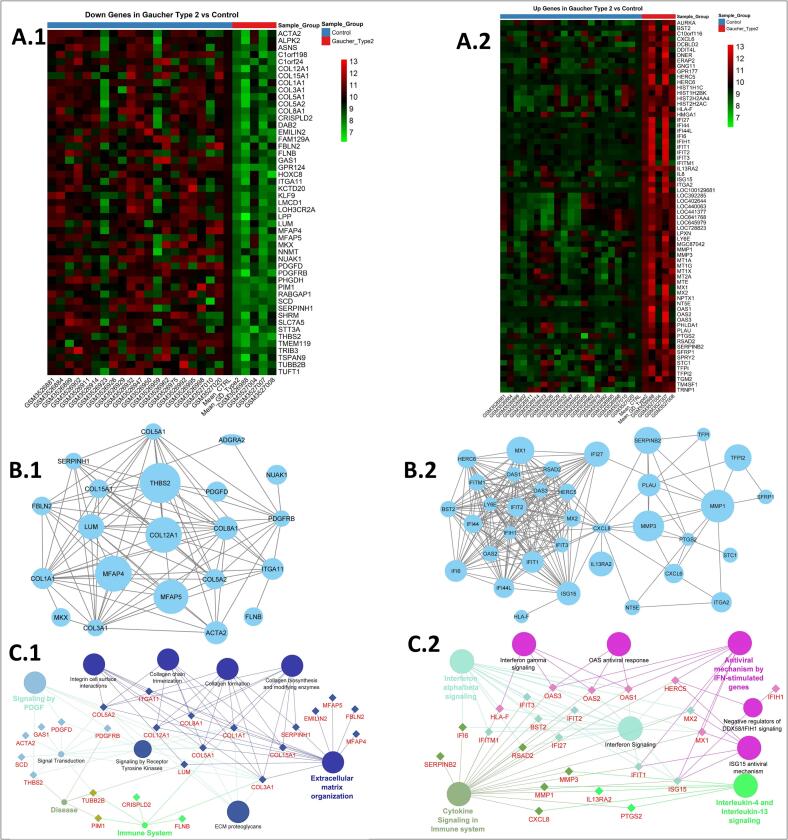
Enriched up and down regulated gene networks in Gaucher type 2. A.1: Down regulated genes. A.2: Up regulated genes. PPI networks and Reactome pathway enrichments. B.1 and C.1, down regulated DEGs; B.2 and C.2, up regulated DEGs.

A heatmap displayed lowly expressed gene profile across patient and control groups (Fig. 1, B.1). The DEGs analysis revealed 66 genes are downregulated in patient samples compared to controls, and used to construct the gene network (28 nodes, 45 edges, protein-protein interaction enrichment, *p* = 3.26e-3). The key hub genes including *TGFBR2, IL1R1, FST, CTSK, PIK3R1, TGFBR3,* and *PLAU* were identified within the network (Fig. 1, C.3). Pathway enrichment analysis showed developmental biology (*p* = 0.5e-2), *TGF-β* signaling (*p* = 1.05e-4), adaptive immunity (*p* = 2.7e-3), metabolism of lipids (*p* = 3.01e-5) and ECM organization (*p* = 0.03e-2) (Fig. 1, E.3). These genes localized to plasma membrane surfaces, external encapsulating structures, and membrane protein complexes (Supplementary, Fig. 2A).

##### 3.1.2.2 Up regulated genes

A heatmap visualized the highly expressed gene pattern across patient and control samples (Fig. 1, B.2). Forty-eight genes were upregulated in patient samples, as determined by LogFC analysis, and were integrated into a gene interaction network with 24 nodes and 37 edges (protein-protein interaction enrichment, *p* = 2.33e-3). The key hub genes within the network were identified as *COL4A1, IGF1, ALDH1B1, KRT7, MYH2, ADA2,* and *HSPB6* (Fig. 1, C.4). These genes were enriched in Platelet degranulation (*p* = 0.05e-1), surfactant metabolism (*p* = 0.1e-2), GPCR signaling (*p* = 0.09), metabolism of proteins (*p* = 0.07), and Phase-1 compound functionalization (p = 0.07e-1) (Fig. 1, E.4), with cellular localization to vesicle lumen, collagen ECM structures, and extracellular vesicles (Supplementary, Fig. 2B).

### 3.2 Gaucher disease type 2 (GD2)

#### 3.2.1 Down regulated genes

**Fig. 3:**
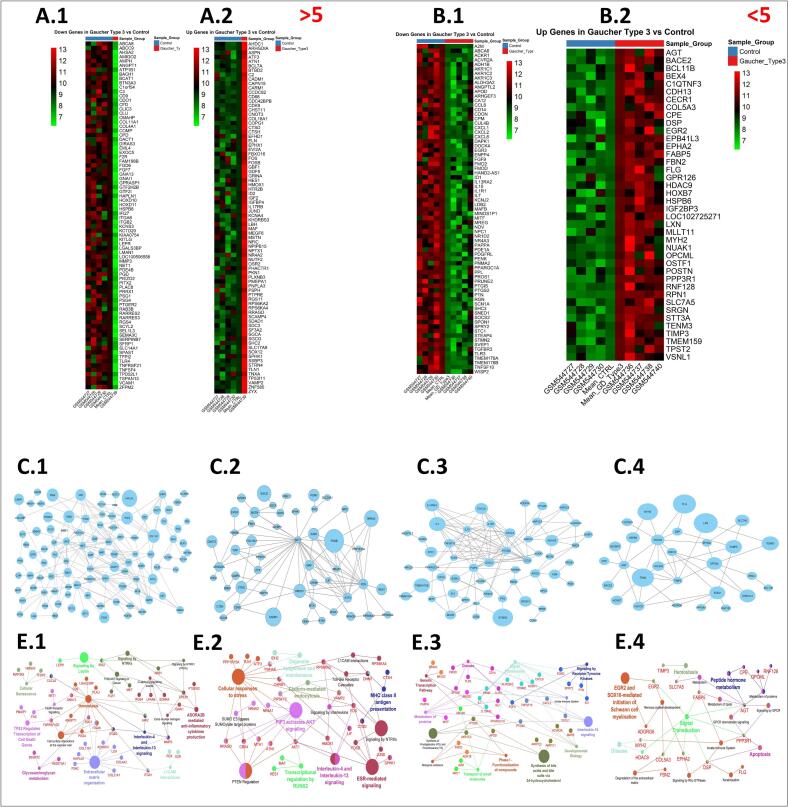
Enriched up and down regulated gene networks in Gaucher type 3. The down and up regulated genes among patients older than 50 years (A.1: Down regulated genes and A.2: Up regulated genes) and younger than 50 years (B.1: Down regulated genes and B.2: Up regulated genes). PPI networks and Reactome pathway enrichments in patients older than 5 years (C.1, and E.1, Down regulated DEGs; C.2 and E.2, Up regulated DEGs). PPI networks and Reactome pathway enrichments in patients younger than 50 years (C.3 and E.3, Down regulated DEGs; C.4 and E.4, Up regulated DEGs).

A heatmap displayed lowly expressed gene profile across patient and control groups (Fig. 2, A.1). A network including 21 nodes and 95 edges was made from 49 down regulated genes based on LogFC analysis (protein-protein interaction enrichment, *p* = 0.29e-6). The key hub genes, such as *COL12A1, MFAP4/5, THBS2, COL8A1, ITGA11, LUM,* and *FBLN2* were identified within the network (Fig. 2, B.1). The network enrichment is related to ECM organization (*p* = 0.03), Receptor tyrosine kinase (*p* = 0.9e-5), *PDGF* signaling (*p* = 0.01e-1), and immune system processes (*p* = 0.007) (Fig. 2, C.1), showing cellular localization to endoplasmic reticulum lumen, collagen ECM, and lysosome (Supplementary, Fig. 3A).

#### 3.2.2 Up regulated genes

A heatmap visualized the highly expressed gene pattern across patient and control samples (Fig. 2, A.2). The DEGs analysis identified 69 up regulated genes forming a dense PPI network (35 nodes, 235 edges; protein-protein interaction enrichment, *p* = 0.16e-8). The key hub genes were identified as *ISG15, MX1/2, OAS1, RSAD2, IFIT1/3, MMP1,* and *CXCL8 (IL8)* within the network (Fig. 2, B.2). The network enrichment was presented the important interleukin-4/13 signaling (*p* = 1.7e-3), *IFN*-mediated antiviral mechanisms (*p* = 0.05e-2), OAS antiviral response (*p* = 0.6e-4), and interferon *α/β/γ* pathways (*p* = 1.11e-4) (Fig. 2, C.2), with predominant localization to plasma membrane, integrin complexes, and Golgi membranes (Supplementary, Fig. 3B).

### 3.3 Gaucher disease type 3 (GD3)

#### 3.3.1 Patients older than 5 years age

##### 3.3.1.1 Down regulated genes

**Fig. 4:**
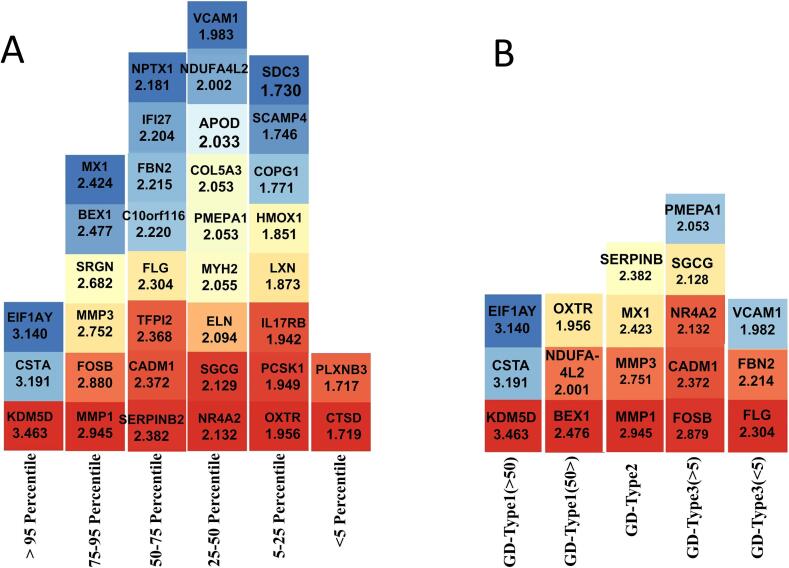
Predicted gene-Specific Markers in Gaucher. A, Distribution of key up regulated genes. B, Up regulated genes (above 95th percentile) uniquely identified in each of the five Gaucher disease subgroups, categorized by disease types and age groups.

To generate the heatmap shown in Fig. 3 A.1, a subset of 80 lowly expressed genes (Top80) was selected from an initial log-transformed expression profile (*n* = 227) identified across patient and control samples. The DEGs analysis showed 227 down regulated genes that were applied to construct a large network based on LogFC analysis (102 nodes, 192 edges; protein-protein interaction enrichment, *p* = 0.9e-5). The key hub genes containing *CXCL8, JAK2, MET, SDC1, CREB1, FZR*, and *FGF7* were found within the network (Fig. 3, C.1). The network was enriched in ECM organization (*p* = 0.01e-3), *p53*-regulated apoptosis (*p* = 1.31e-4), hemostasis (*p* = 3.01e-4), anti-inflammatory cytokine (*p* = 0.02), and *NTRK* signaling (*p* = 0.03) pathways (Fig. 3, E.1), with localization to ECM structures, membrane surfaces, and lipid rafts (Supplementary, Fig. 4A).

##### 3.3.1.2 Up regulated genes

Figure 3 A.2 presented a heatmap generated from a curated subset of 80 highly expressed genes (Top80), selected from an initial log-transformed expression dataset (*n* = 122) profiled across patient and control samples. Differential expression analysis found 122 up regulated genes to make a PPI network with 50 nodes and 93 edges (protein-protein interaction enrichment, *p* = 2.06e-3). The key hub genes, including *AKT1, FOS, ATF3, FOSB, JUND, HMOX1*, and *MAF* were determined within the network (Fig. 3, C.2). The network enrichment analysis revealed MHC class II antigen presentation (*p* = 5.01e-4), cellular stress responses (p = 0.01e-4), *PI3K/AKT* signaling (*p* = 1.01e-3), *PTEN* regulation (*p* = 0.001e), and interleukin-4/13 pathways (p = 0.01) (Fig. 3, E.2), localized to cell-cell junctions, endosome membranes, and synapses (Supplementary, Fig. 4B).

#### 3.3.2 Patients younger than 5 years age

##### 3.3.2.1 Down regulated genes

A heatmap displayed lowly expressed gene profile across patient and control groups (Fig. 3, B.1). The DEGs analysis revealed 73 down regulated genes that were applied to form a network with 52 nodes and 123 edges (protein-protein interaction enrichment, *p* = 6e-8). The *PTGS2, IL15, CXCL1, CCL8, FGF9, IL1R1,* and *CXCL2* were identified as key hub genes within the network (Fig. 3, C.3). Receptor tyrosine kinase signaling (p = 0.001), interleukin-10 (*p* = 3.51 e-6), Synthesis of prostaglandins and thromboxane (*p* = 9.01 e-6) and protein metabolism (*p* = 0.31 e-3) pathways (Fig. 3, E.3) were found on the network enrichment, localizing to extracellular vesicles, nucleus, and cytoplasmic vesicles (Supplementary, Fig. 5 A).

##### 3.3.2.2 Up regulated genes

A heatmap was generated to visualize the highly expressed gene profile across patient and control samples (Fig. 3, B.2). A network including 29 nodes and 46 edges (protein-protein interaction enrichment, *p* = 3.13e-2) was constructed from 40 up regulated genes. The key hub genes such as *POSTN, TIMP3, AGT, OPCML, FBN2, EGR2,* and *CDH13* were observed within the network (Fig. 3, C.4). The network enrichment demonstrated nervous system development (*p* = 0.2 e-3), Schwann cell myelination (*EGR2/SOX10*) (p = 1.01 e-4), apoptosis (*p* = 0.22 e-3), and GPCR/peptide hormone signaling pathways (*p* = 0.03) (Fig. 3, E.4), localized to extracellular space, plasma membrane, and cytoplasm (Supplementary, Fig. 5B).

A comprehensive overview of differentially expressed genes, enriched pathways, and cellular components across Gaucher disease subtypes and age groups is summarized in Table 2.

**Table 2:** Study data.

Gaucher Type	Age Group (Year)	Upregulated Genes (n)/Hub	Downregulated Genes (n)/Hub	Upregulated PPI Network (Nodes/Edges, p-value)	Downregulated PPI Network (Nodes/Edges, p-value)	Enriched Pathways (Downregulated Genes)	Enriched Pathways (Upregulated Genes)	Cellular Components (Downregulated Genes)	Cellular Components (Upregulated Genes)
1	> 50	**57**/ TP53, HNRNPL, SF3A2, YWHAE, ATN1, SOD3, MAZ, CBS	**58**/ IL6, LEPR, PPARGC1A, STAT4, IL7R, TFPI2, ADAMTS1 NAMPT	(28 / 40, p = 8.66e-4)	(24 / 42, *p* = 1.21e-4)	cytokine-mediated immune signaling, cellular response to stimuli, Immune system, Signaling by interleukins, NGF-stimulated transcription, TRKA signal transduction	mRNA splicing, Membrane trafficking, nuclear receptor signaling, protein metabolism, PTEN regulation, activation of BH3-only proteins	Cytoplasm, Intracellular organelle	Vesicles, Extracellular space, Plasma membrane
	< 50	**48**/ COL4A1, IGF1, ALDH1B1, KRT7, MYH2, ADA2, and HSPB6	**66**/ TGFBR2, IL1R1, FST, CTSK, PIK3R1, TGFBR3, and PLAU	(24 / 37, p = 2.33e-3)	(28 / 45, p = 3.26e-3)	Developmental biology, TGFB signaling, Adaptive immune system, ECM organization, metabolism of lipids	Protein-protein interaction at synapses, Surfactant metabolism, GPCR signaling, Platelet degranulation	External side of plasma membrane, External encapsulating structures, Membrane protein complexes	Vesicle lumen, Collagen extracellular matrix, Extracellular vesicles
2		**69**/ ISG15, MX1/2, OAS1, RSAD2, IFIT1/3, MMP1, and CXCL8	**49**/ COL12A1, MFAP4/5, THBS2, COL8A1, ITGA11, LUM, and FBLN2	(35 / 235, p = 0.16e-8)	(21 / 95, p = 0.29e-6)	ECM organization, Immune system, PDGF signaling, Receptor tyrosine kinase	Interleukin 4/13, Antiviral mechanisms (IFN), Interferon alpha/beta signaling, OAS antiviral response	ER lumen, Collagen ECM, Lysosome, Cytoskeleton, Cytoplasm	Plasma membrane, Integrin alpha2/beta1 complex, Nucleoplasm, Golgi membrane
3	> 5	**122**/ AKT1, FOS, ATF3, FOSB, JUND, HMOX1, and MAF	**227**/ CXCL8, JAK2, MET, SDC1, CREB1, FZR, and FGF7	(50 / 93, p = 2.06e-3)	(102/192, p = 0.9e-5)	ECM organization, TP53-regulated transcription, Hemostasis, NTRK signaling, anti-inflammatory cytokine	MHC class II antigen presentation, Cellular response to stress, PIP3/AKT signaling, Interleukin (4/13)	ECM, External side of membrane, Membrane raft	Cell-cell junctions, Endosome membrane, Chromatin, Synapse
	< 5	**57**/ POSTN, TIMP3, AGT, OPCML, FBN2, EGR2, and CDH13	**109**/ PTGS2, IL15, CXCL1, CCL8, FGF9, IL1R1, and CXCL2	(29 / 46, p = 3.13e-2)	(52 / 123, *p* = 0.06e-6)	Receptor tyrosine kinase signaling, Interleukin 10 signaling, Protein metabolism, Synthesis of prostaglandins and thromboxane	Nervous system development, Schwann cell myelination, Apoptosis, GPCR signaling	Extracellular vesicle, Nucleus, Cytoplasmic vesicle	Extracellular space, Plasma membrane, Cytoplasm

### 3.4 Potential subtype- and age-specific gene markers

A total of 36 up regulated genes were identified across the five patient subgroups, as shown in Fig. 4A. Notably, *KDM5D* (Fold 3.463), *CSTA* (Fold 3.191), and *EIF1AY* (Fold 3.140) emerged consistently in the percentile >95 % for all the GD. The subgroup-specific screening markers were also predicted. The *KDM5D, CSTA*, and *EIF1AY* were exclusively present in GD1 patients aged >50 years, while the *BEX1*, *NDUFA4L2*, and *OXTR* were found in GD1 patients aged <50 years. In the GD2 patients, the *MMP1*, *MMP3*, *MX1*, and *SERPINB* were suggested as the screening markers. Furthermore, for the GD3 patients aged >5 years, the *FOSB, CADM1, NR4A2, SGCG,* and *PMEPA1*, and for GD3 patients aged <5 years, the *FLG*, *FBN2*, and *VCAM1* enabling the delineation of potential expression signatures, were found (Fig. 4B).

## 4 Discussion

The present study provides a comprehensive transcriptomic analysis of differentially expressed genes (DEGs) in Gaucher disease (GD) subtypes 1, 2, and 3, uncovering critical molecular pathways and cellular components associated with disease progression and severity. Through the integration of differential gene expression analysis, protein-protein interaction (PPI) network construction, and pathway enrichment studies, our findings highlighted the molecular perturbations underlying the pathophysiology of GD and their potential implications in therapeutic interventions.

One of the key findings in GD1 was the age-dependent variations in gene expression profiles [16,17]. In individuals aged >50 years, some down regulated genes were involved in cytokine-mediated immune signaling, *NGF*-stimulated transcription, and *TRKA* signal transduction. This aligns with previous studies indicating immune dysregulation in GD patients [[18], [19], [20]]. Notably, the upregulation of *TP53* and *SOD3* suggests compensatory oxidative stress mitigation, consistent with studies linking glucosylceramide accumulation to mitochondrial dysfunction and *p53*-mediated apoptosis [21]. The up regulated genes in this subtype were predominantly enriched in membrane trafficking, protein metabolism, and nuclear receptor signaling, indicating potential cellular stress responses following lysosomal dysfunction. These findings are consistent with studies highlighting alterations in protein processing pathways in GD. The accumulation of misfolded proteins and subsequent endoplasmic reticulum stress have been implicated in GD, underscoring the relevance of these upregulated pathways [22,23]. In contrast, younger individuals with GD1 (<50 years) exhibited differential expression patterns associated with *TGF-β* signaling, adaptive immunity, and extracellular matrix (ECM) organization pathways [24,25]. The prominence of ECM-related genes suggests a role for altered matrix homeostasis in GD1 pathology, active remodeling and inflammatory responses. Similar patterns have been noted in examining bone pathology in GD, where disruptions in ECM components contribute to skeletal abnormalities [26,27]. *TGF-β* signaling disruption (*TGFBR2, FST*) and ECM dysregulation (*COL4A1, PLAU*) provide mechanistic insights into early GD1 complications. The downregulation of *PLAU* aligns with impaired fibrinolysis observed in GD1-associated bone crises, while *COL4A1* overexpression may reflect aberrant angiogenesis, a feature increasingly recognized in GD1 vasculopathy. These findings extend recent transcriptomic evidence of matrix metalloproteinase dysregulation in GD fibroblasts, suggesting age-specific ECM homeostasis thresholds [28,29]. The downregulation of genes involved in ECM maintenance may underlie the bone fragility observed in GD1 patients [19,30]. While GD1 is traditionally considered non-neuropathic, emerging evidence in upregulation of genes in this group suggests subtle neurological manifestations. The upregulation of synaptic-related genes (*YWHAE, ATN1*) may reflect compensatory mechanisms or early neuronal changes [31,32].

In GD2, a more severe and early-onset form of the disease, there is significant dysregulation in genes associated with ECM organization, PDGF signaling, and immune system processes. These pathways are crucial for neuronal development and maintenance, and their disruption aligns with the severe neurological deterioration characteristics of GD2 [33,34]. Our findings have also reported significant alterations in ECM-related genes (*COL12A1, MFAP4/5, THBS2, COL8A1, ITGA11, LUM, FBLN2*) in neuropathic GD, supporting previous studies [35]. The ECM is crucial for neuronal support, synaptic stability, and tissue repair; its disruption likely contributes to the severe neurological deterioration and tissue damage observed in GD2. This ECM dysregulation may also impair cell-to-cell and cell-to-matrix communication, further destabilizing neuronal networks and accelerating neurodegeneration. Moreover, the study results showed upregulation of genes involved in interleukin-4/13 signaling, interferon-mediated antiviral defense, and other innate immune pathways (*ISG15, MX1/2, OAS1, RSAD2, IFIT1/3, MMP1, CXCL8*) [36,37]. This suggests a heightened inflammatory state in GD2, corroborating reports of increased cytokine activity in neuropathic GD forms. The pronounced neuroinflammation may exacerbate neuronal damage, contributing to the rapid disease progression observed in GD2 patients [38,39]. The prominent involvement of lysosomal and Golgi membrane-associated genes supports the notion of a fundamental defect in intracellular trafficking and lysosomal enzyme processing, which aligns with the underlying etiology of GD [40]. The accumulation of glucocerebroside due to deficient glucocerebrosidase activity disrupts normal lysosomal function, leading to cellular toxicity, impaired autophagy, and secondary activation of stress and inflammatory pathways. These findings reinforce previous reports that implicate chronic inflammation and dysregulated immune responses in the rapid progression of GD2.

Similarly, in GD3, the gene expression landscape was different between age groups. In patients older than five years, the downregulation of genes involved in ECM organization (*CXCL8, SDC1, FGF7*), *p53*-regulated apoptosis (*JAK2, MET, CREB1*), and *NTRK* signaling points toward a disrupted balance between cell survival and programmed cell death, potentially contributing to neuronal loss. These findings are consistent with studies indicating that disruptions in ECM integrity and apoptotic pathways contribute to the chronic neuronopathic features of GD3 [41]. The downregulation of neurotrophic signaling pathways may further impair neuronal survival and function [42,43]. Up regulated genes in this group were linked to MHC class II antigen presentation (*FOS, ATF3, JUND*), cellular stress responses (*HMOX1, MAF*), and *PI3K/AKT* signaling (*AKT1*) [[44], [45], [46]]. These results suggest a potential compensatory mechanism involving immune modulation and survival signaling. In contrast, the younger GD3 subgroup (<5 years) displayed the enriched receptor tyrosine kinase signaling (*PTGS2, FGF9, IL1R1*), interleukin-10, and protein metabolism pathways through the downregulated genes. The *PI3K/AKT* pathway, in particular, is recognized for its neuroprotective and anti-apoptotic effects, indicating a possible attempt by affected cells to counteract neurodegenerative processes. These pathways are vital for neuronal growth and function, and their suppression may contribute to the early-onset neurological impairments seen in younger GD3 patients [47,48]. Comparable gene expression alterations have been observed in early-onset neuronopathic GD cases, reinforcing the relevance of these pathways. The up regulated genes in this group were associated with nervous system development (*POSTN, FBN2, EGR2*), Schwann cell myelination pathways (*EGR2/SOX10*), and apoptosis (*TIMP3, AGT*), reinforcing the critical involvement of neurodevelopmental defects and neuroinflammation in GD3 pathology. The activation of myelination pathways may reflect attempts at remyelination or compensatory responses to demyelination, a hypothesis supported by recent findings in neuronopathic GD models. The involvement of GPCR/peptide hormone signaling also suggests alterations in neuroendocrine communication, which could contribute to the complex behavioral and neurological phenotype forms of GD3 [49,50].

A notable aspect of our study is the alignment of molecular findings with the clinical heterogeneity observed in GD. The differential expression of genes related to immune regulation, neuronal function, and ECM integrity across subtypes and age groups suggests that distinct molecular mechanisms drive disease severity and progression [51,52]. This is particularly relevant given the variability in neurological involvement between GD1 (non-neuronopathic), GD2 (acute neuronopathic), and GD3 (subacute neuronopathic). Our results emphasized that therapeutic strategies targeting immune dysregulation, lysosomal homeostasis, and neurotrophic signaling may be more effective if tailored according to disease subtype and patient age. Comparison with previous transcriptomic and proteomic studies on GD revealed both concordant and novel findings [34,51,52] on all of GD subtypes. It explained the differential clinical manifestations based on the age-specific and subtype-specific variations. Additionally, the identification of novel DEGs and pathway perturbations provided potential therapeutic targets. However, the increase of sample sizes (GSM) and identification of clinicopathological characteristics in datasets can improve the statistical power for DEG identification. Validation is necessary through independent patient cohorts and functional assays to confirm the biological relevance of identified genes and pathways. Future studies incorporating single-cell transcriptomic and proteomic analyses may provide a more granular understanding of cell-type-specific contributions to GD pathophysiology.

The percentile-based stratification framework implemented in this study enabled precise prioritization of differentially expressed genes across diverse subtypes and age groups in Gaucher disease, offering novel insights into subtype-specific molecular landscapes. Genes residing in the top 5 % of upregulation (>95th percentile) were proposed to have enhanced biological relevance due to their expression magnitudes. Among these, *KDM5D, CSTA*, and *EIF1AY* stood out as common high-expression genes, especially in GD1 patients over 50 years old. It suggests a potential central role in GD pathophysiology, perhaps linked to age-related immune dysregulation or epigenetic remodeling [53]. More importantly, subgroup-specific marker genes were identified that were both highly expressed and uniquely present. For instance, in GD1 patients (<50 years), *BEX1, NDUFA4L2,* and *OXTR* were exclusively up regulated, suggesting roles in mitochondrial function, stress response, and neuroendocrine signaling pathways previously implicated in neuronopathic manifestations of GD [[54], [55], [56]]. In GD2 patients, high expression of matrix remodeling genes such as *MMP1* and *MMP3*, along with interferon-related genes like *MX1*, might reflect extracellular matrix turnover characteristic of the more aggressive GD2 phenotype [57]. In GD3 patients over 5 years old, genes such as *FOSB, NR4A2*, and *PMEPA1* point toward disrupted transcriptional regulation and *TGF-β* signaling, aligning with developmental and neurological deficits often seen in this subgroup [58]. Conversely, in the under-five GD3 group, the exclusive expression of *FLG, FBN2,* and *VCAM1* hints at vascular involvement and structural extracellular matrix disruption in early disease stages [59,60]. These highly specific and biologically diverse gene signatures strengthen the argument for tailored biomarker panels that reflect age-dependent disease types. Such refined panels may improve screening specificity and aid in early subtype distinction.

The study data were based on cultured skin fibroblasts. The fibroblasts, however, are a useful patient-derived model for assessing core cellular perturbations related to *GBA1* deficiency including lysosomal dysfunction, ER stress, autophagy, and extracellular matrix remodeling but the study of other cells such as macrophages is suggested to evaluate the signaling pathways and gene patterns based on their roles in different tissues.

In conclusion, this study presented an in-depth transcriptomic dissection of Gaucher disease (GD) subtypes, integrating differential expression analysis, network modeling, pathway enrichment, and percentile-based prioritization of up regulated genes. By combining conventional DEG identification with stratification of gene expression magnitudes, the results not only identified key signaling and cellular processes but also proposed highly specific candidate biomarkers to age- and subtype-defined GD patient groups. The identification of uniquely up regulated genes within the top percentile for each subgroup provides a strong foundation for the development of targeted diagnostic panels. These findings reinforce the need for precision medicine approaches that consider the molecular heterogeneity of GD and open new avenues for biomarker-guided screening and personalized intervention strategies. Future experimental validation and clinical correlation studies are essential to translate these molecular signatures into effective tools for disease monitoring and therapeutic decision-making.

## CRediT authorship contribution statement

**Mohammad Elahimanesh:** Writing – original draft, Visualization, Validation, Software, Methodology, Investigation, Data curation. **Reza Ganjali:** Writing – original draft, Resources, Methodology. **Mohammad Najafi:** Supervision, Funding acquisition, Conceptualization.

## Consent for publication

Not applicable.

## Ethics approval and consent to participate

Not applicable.

## Human and animal rights

No animals/humans were used for this review.

## Funding

No.

## Declaration of competing interest

The authors declare no conflict of interest, financial or otherwise.

## Data Availability

The data generated and analyzed during the current study are available from the corresponding author on reasonable request.
