# Supplementary material for: Transcriptomic signatures in Gaucher disease subtypes: A systems biology perspective
Source: Mol Genet Metab Rep. 2025 Nov 13;45:101274. doi: 10.1016/j.ymgmr.2025.101274 (PMC12663027; doi:10.1016/j.ymgmr.2025.101274)
Supplement: Supplementary material [file mmc1.pptx]

## Slide 1
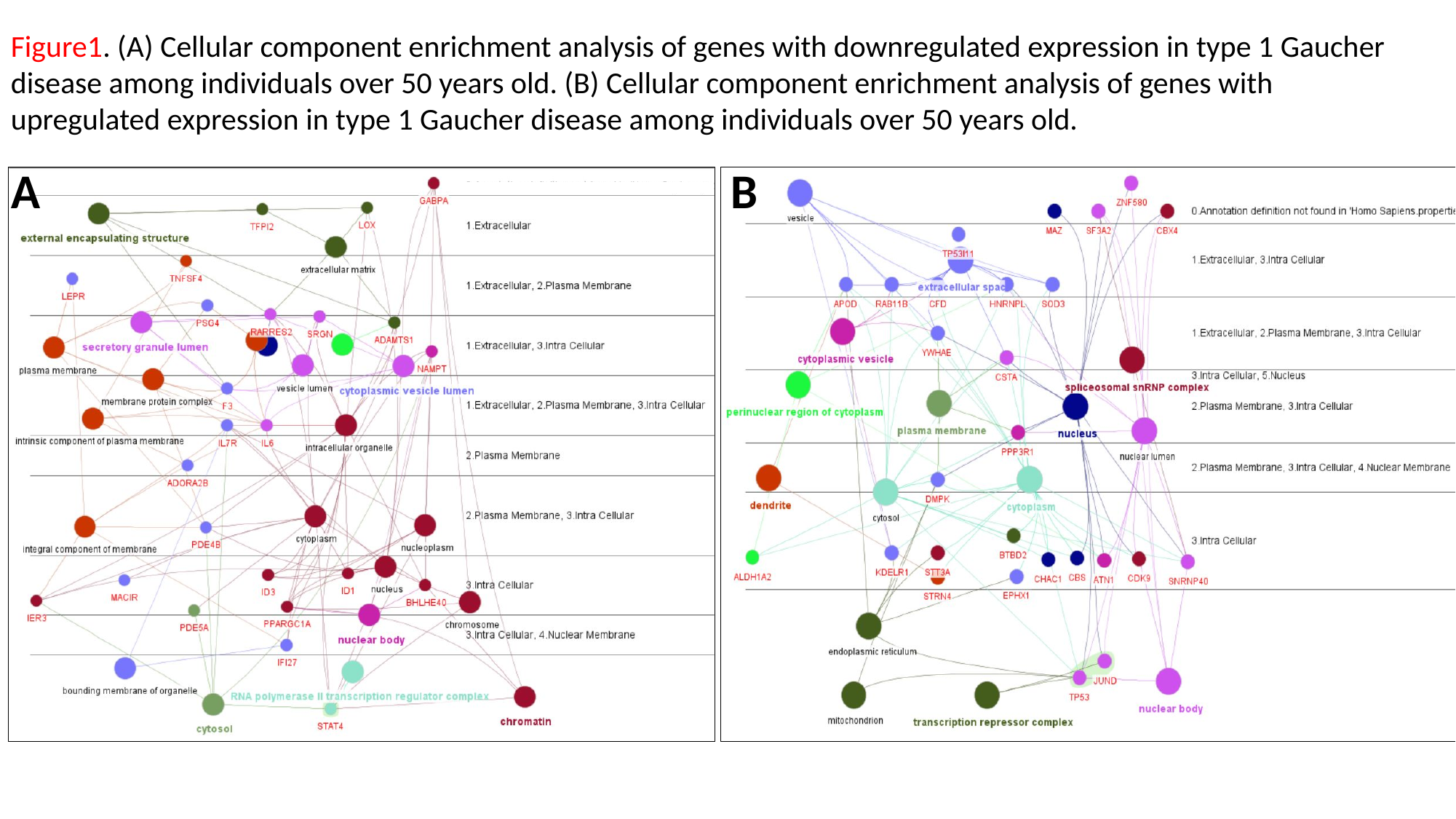

Figure1. (A) Cellular component enrichment analysis of genes with downregulated expression in type 1 Gaucher disease among individuals over 50 years old. (B) Cellular component enrichment analysis of genes with upregulated expression in type 1 Gaucher disease among individuals over 50 years old.
A
B
figure3

## Slide 2
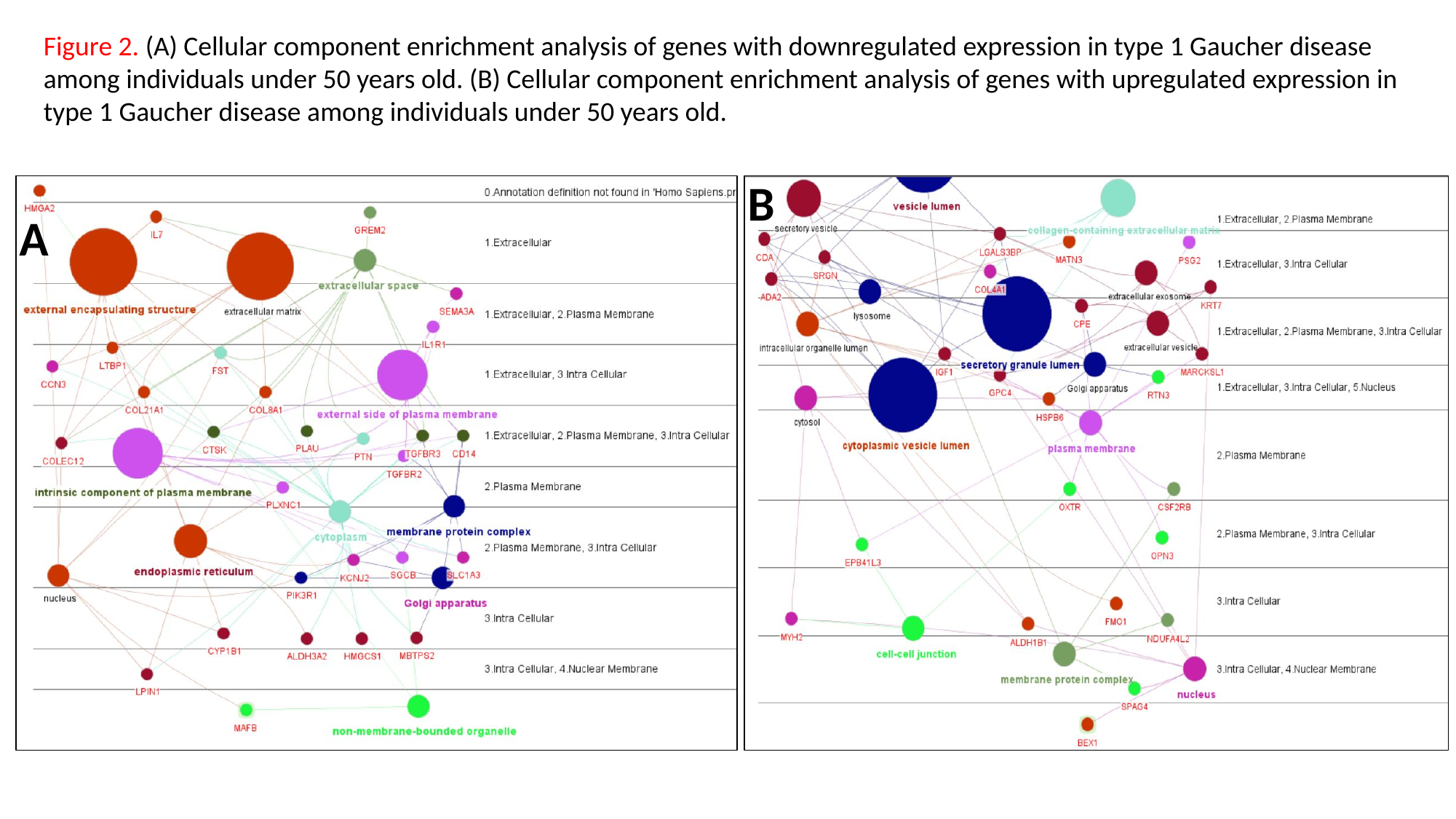

Figure 2. (A) Cellular component enrichment analysis of genes with downregulated expression in type 1 Gaucher disease among individuals under 50 years old. (B) Cellular component enrichment analysis of genes with upregulated expression in type 1 Gaucher disease among individuals under 50 years old.
B
figure5
A

## Slide 3
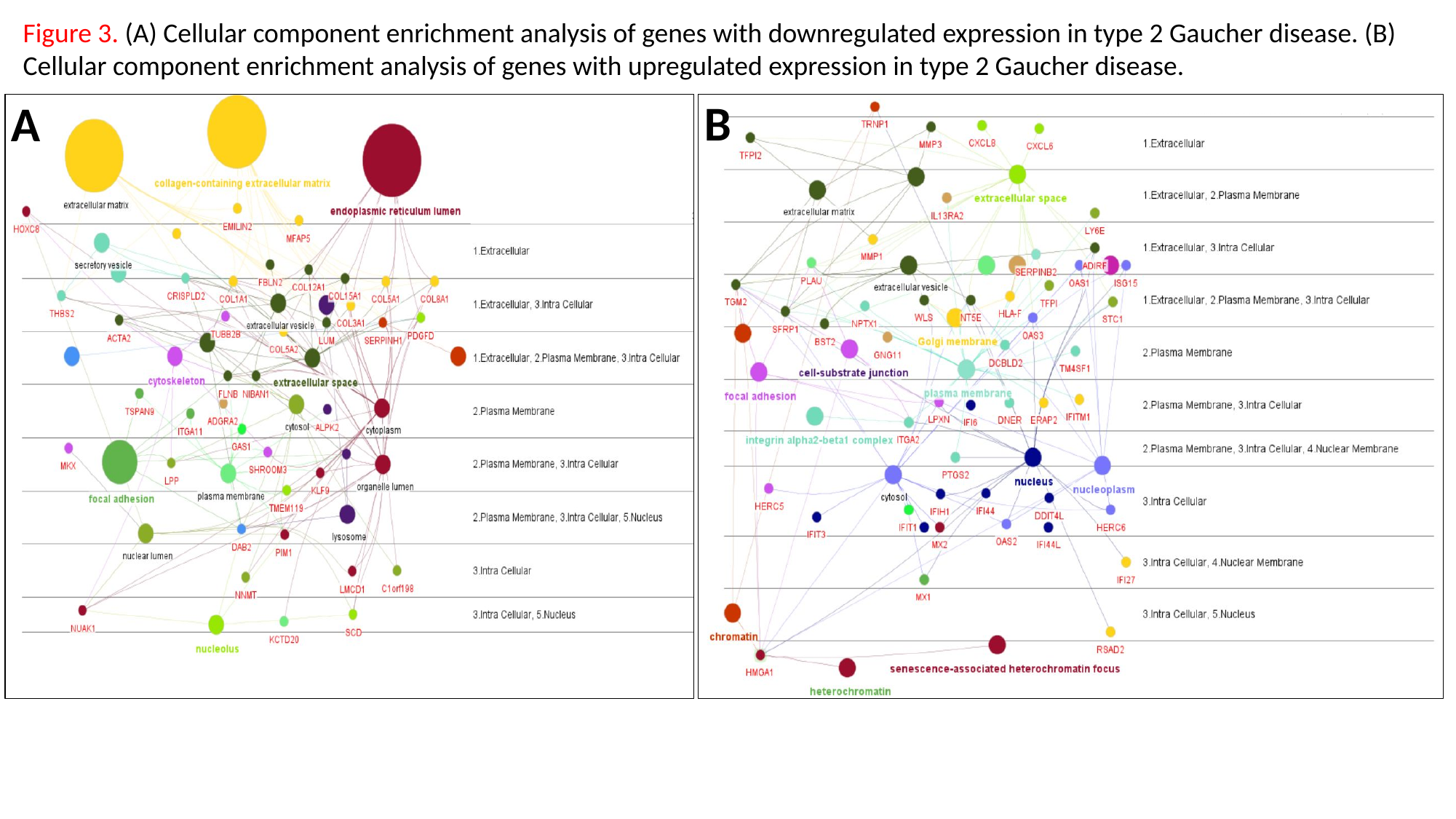

Figure 3. (A) Cellular component enrichment analysis of genes with downregulated expression in type 2 Gaucher disease. (B) Cellular component enrichment analysis of genes with upregulated expression in type 2 Gaucher disease.
B
A
figure8

## Slide 4
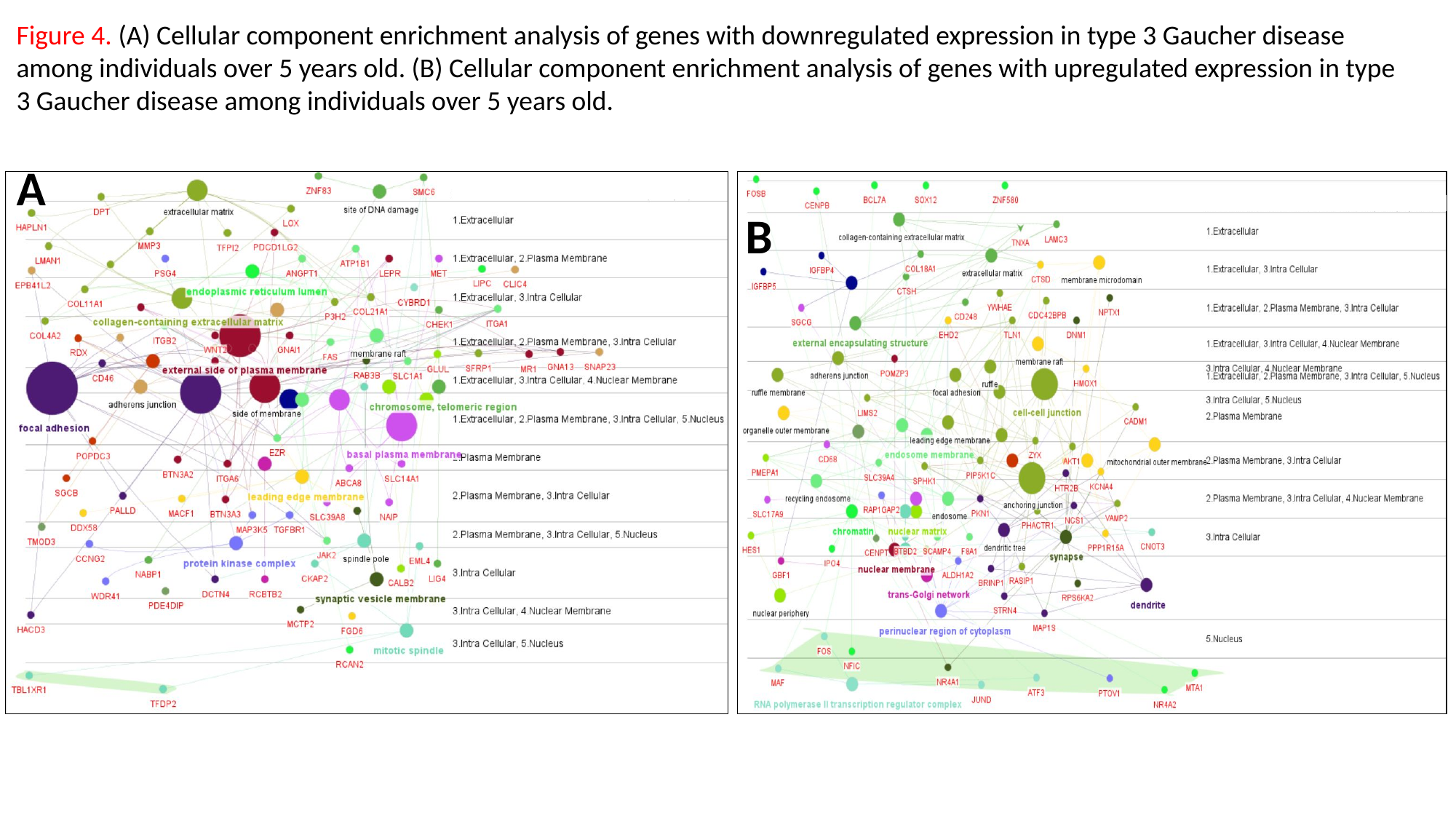

Figure 4. (A) Cellular component enrichment analysis of genes with downregulated expression in type 3 Gaucher disease among individuals over 5 years old. (B) Cellular component enrichment analysis of genes with upregulated expression in type 3 Gaucher disease among individuals over 5 years old.
A
figure11
B

## Slide 5
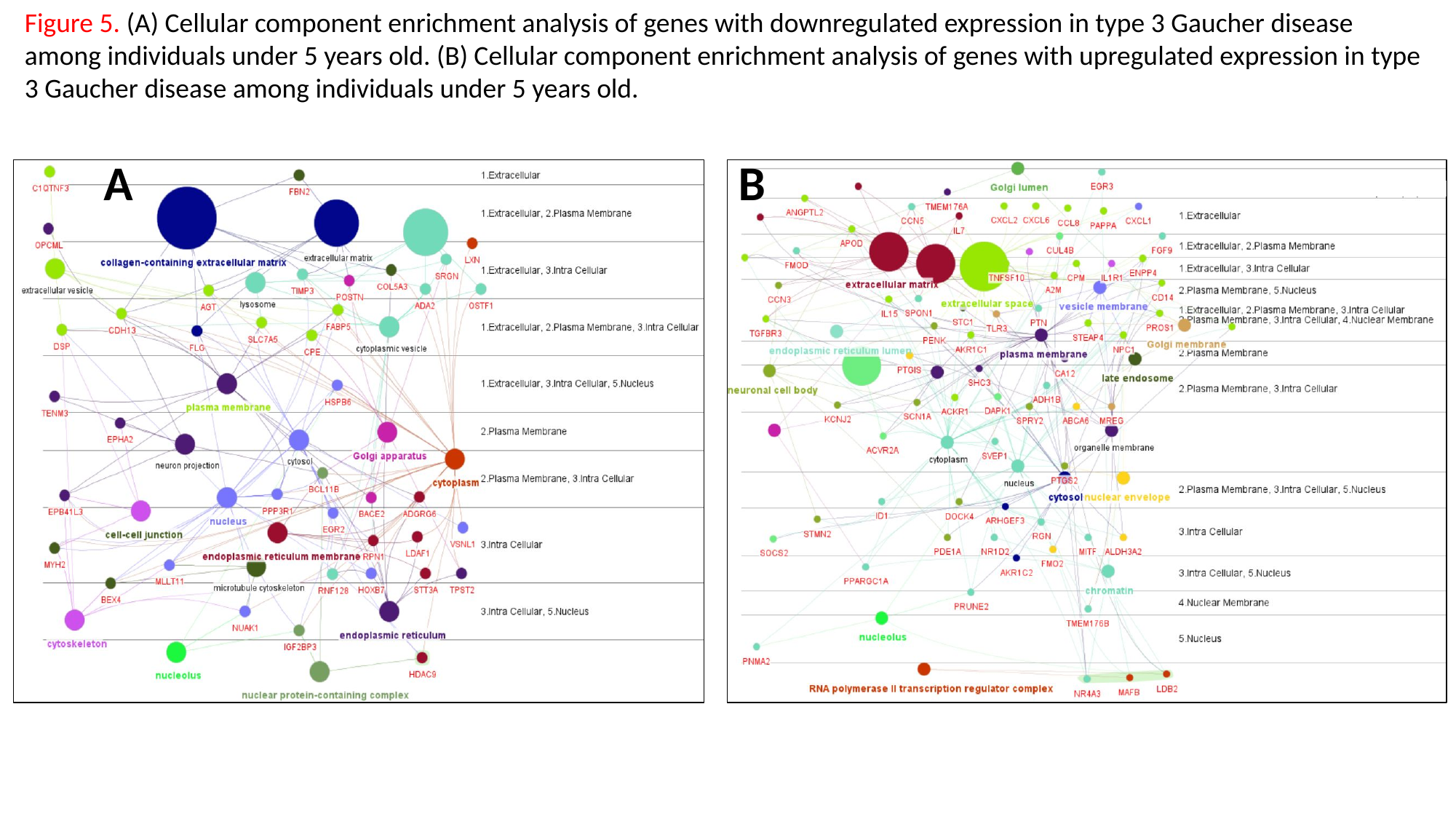

Figure 5. (A) Cellular component enrichment analysis of genes with downregulated expression in type 3 Gaucher disease among individuals under 5 years old. (B) Cellular component enrichment analysis of genes with upregulated expression in type 3 Gaucher disease among individuals under 5 years old.
A
B
figure13
